# Up-Regulation of the ATP-Binding Cassette Transporter A1 Inhibits Hepatitis C Virus Infection

**DOI:** 10.1371/journal.pone.0092140

**Published:** 2014-03-19

**Authors:** Simone Bocchetta, Patrick Maillard, Mami Yamamoto, Claire Gondeau, Florian Douam, Stéphanie Lebreton, Sylvie Lagaye, Stanislas Pol, François Helle, Wanee Plengpanich, Maryse Guérin, Maryline Bourgine, Marie Louise Michel, Dimitri Lavillette, Philippe Roingeard, Wilfried le Goff, Agata Budkowska

**Affiliations:** 1 Unité Hépacivirus et Immunité Innée, CNRS, UMR3569, Paris, France; 2 Dipartimento di Medicina Translazionale, Università del Piemonte Orientale, “Amedeo Avogadro”, Novara, Italy; 3 Department of Biochemistry, Nihon University School of Medicine, Tokyo, Japan; 4 INSERM U1040, Institut de Recherche en Biothérapie, Hôpital Saint-Eloi, Montpellier, France; 5 Groupe de Recherche Dynamique Microbienne et Transmission virale, UMR CNRS 5557, Ecologie Microbienne, Villeurbanne, France; 6 Unité Trafic Membranaire et Pathogenèse, Institut Pasteur, Paris, France; 7 Unité d’Hépatologie, AP-HP, Groupe Hospitalier Cochin-Hôtel Dieu, Paris, France; 8 Equipe Cycle Cellulaire, Régénération et Hépatopathies, INSERM U1016, CNRS UMR8104, Institut Cochin, Paris, France; 9 Laboratoire de Virologie, CHU Sud Amiens, Centre de Biologie Humaine, Amiens, France; 10 Dyslipidemia, Inflammation and Atherosclerosis in Metabolic Diseases, INSERM UMRS939, Paris, France; 11 Endocrinology and Metabolism Unit, Department of Medecine, Chulalongkorn University and King Chulalongkorn Memorial Hospital, Thai Red Cross Society, Bangkok, Thailand; 12 Pathogénèse des Virus de l′Hépatite B, INSERM U845, Institut Pasteur, Paris, France; 13 INSERM U966, Université François-Rabelais and CHRU de Tours, Tours, France; Institut Pasteur, France

## Abstract

Hepatitis C virus (HCV) establishes infection using host lipid metabolism pathways that are thus considered potential targets for indirect anti-HCV strategies. HCV enters the cell via clathrin-dependent endocytosis, interacting with several receptors, and virus-cell fusion, which depends on acidic pH and the integrity of cholesterol-rich domains of the hepatocyte membrane. The ATP-binding Cassette Transporter A1 (ABCA1) mediates cholesterol efflux from hepatocytes to extracellular Apolipoprotein A1 and moves cholesterol within cell membranes. Furthermore, it generates high-density lipoprotein (HDL) particles. HDL protects against arteriosclerosis and cardiovascular disease. We show that the up-regulation of ABCA1 gene expression and its cholesterol efflux function in Huh7.5 hepatoma cells, using the liver X receptor (LXR) agonist GW3965, impairs HCV infection and decreases levels of virus produced. ABCA1-stimulation inhibited HCV cell entry, acting on virus-host cell fusion, but had no impact on virus attachment, replication, or assembly/secretion. It did not affect infectivity or properties of virus particles produced. Silencing of the ABCA1 gene and reduction of the specific cholesterol efflux function counteracted the inhibitory effect of the GW3965 on HCV infection, providing evidence for a key role of ABCA1 in this process. Impaired virus-cell entry correlated with the reorganisation of cholesterol-rich membrane microdomains (lipid rafts). The inhibitory effect could be reversed by an exogenous cholesterol supply, indicating that restriction of HCV infection was induced by changes of cholesterol content/distribution in membrane regions essential for virus-cell fusion. Stimulation of ABCA1 expression by GW3965 inhibited HCV infection of both human primary hepatocytes and isolated human liver slices. This study reveals that pharmacological stimulation of the ABCA1-dependent cholesterol efflux pathway disrupts membrane cholesterol homeostasis, leading to the inhibition of virus–cell fusion and thus HCV cell entry. Therefore besides other beneficial roles, ABCA1 might represent a potential target for HCV therapy.

## Introduction

Hepatitis C virus (HCV) infection affects 3% of the world population and is major cause of chronic liver disease with severe hepatic consequences such as steatosis, cirrhosis and hepatocarcinoma. Recently, numerous direct acting anti-viral drugs (DDA) have been introduced, which target essential viral functions. These new treatments represent a significant step forward compared to standard Pegylated IFN-α-ribavirin therapy. DDA are mainly inhibitors of NS3/NS4 HCV protease, and others drugs are under development that target the NS5B polymerase or NS5A that also play essential roles in HCV replication [1]. However, these DDA still have side effects and induce the manifestation of drug-resistance [2]. Novel treatments targeting host cell molecules involved in various steps of the HCV life cycle (such as cyclophilin A, microRNA-122, or phosphatidylinositol-4-kinase III alpha) have been proposed for novel anti-HCV approaches (and these are called “indirect acting anti-viral drugs”, IAAD), to prevent the onset of antiviral resistance and to cure infection with all HCV genotypes [1], [3].

HCV is an enveloped virus of the *Flaviviridae* family (genus *Hepacivirus*) with a single-stranded positive RNA genome of 9.6 kb. The viral genome encodes a polyprotein of 3,000 amino acids, which is cleaved by the host and viral proteases into structural proteins (the capsid protein and two envelope glycoproteins E1 and E2), P7 and several non-structural proteins (NS2, NS3, NS4A, NS4B, NS5A and NS5B) involved in genome replication and virus assembly [3], [4].

Lipids and lipoproteins are essential for the HCV life cycle [5], [6], [7]. HCV has developed lipoprotein-dependent mechanisms for cell entry [8], [9], [10], replication regulated by fatty acids [11], virus morphogenesis that is linked to lipid droplets [12] and the assembly and release of infectious virions *via* the VLDL (very low density lipoprotein) formation and secretion pathway [13], [14]. Consequently, HCV circulates in the plasma of infected patients in association with VLDL and LDL (low-density lipoprotein), forming lipo-viral particles (LVPs) [15], [16].

The relationships between lipid metabolism and HCV are complex and intriguing. The expression of host genes involved in biosynthesis, degradation or transport of intracellular lipids is altered upon HCV infection [17], [18]. Steatosis and insulin resistance associated with the metabolic syndrome increase fibrosis progression and reduce the response to the IFN-α-ribavirin treatment. Moreover, a high baseline LDL level has been shown to be the best predictor of a sustained virologic response, whereas low lipid levels correlate with steatosis, progressing fibrosis and non-response to treatment [19].

Altogether, these observations reflect the important role of lipids in the HCV life cycle. Therefore, host factors involved in cholesterol/lipid metabolism might represent potential targets for HCV strategies, with only limited possibilities for escape mutations to develop [20], [21] and allowing treatment of patients infected with genotype 3 HCV [1].

Cholesterol is an important structural component of biological membranes and is essential for the uptake of many viruses. HCV cell entry requires cholesterol homeostasis and intact cholesterol-rich membrane microdomains [22]. Indeed perturbation of the alignment/packaging of cholesterol in lipid membranes increases the energy barrier required for virus-cell entry *via* fusion mechanisms [23].

Hepatocytes play a vital role in cholesterol homeostasis, acquiring cholesterol by synthesis *via* the mevalonate pathway or by LDL-R mediated endocytosis. Cholesterol is exported from hepatocytes together with triglycerides through the VLDL secretion pathway [24]. However, a major regulator of cellular cholesterol and phospholipid homeostasis is the ABCA1 transporter. ABCA1 is an integral trans-membrane protein that moves phospholipids and free cholesterol across the cell membrane to combine them with lipid-free ApoA1, which is also synthesised in the liver, to form nascent HDL particles [25], [26]. ABCA1 is highly expressed in the liver and tissue macrophages. Nevertheless, the liver ABCA1 pathway appears to generate most (70–80%) plasma HDL [27]. ABCA1 exports cholesterol exclusively at the cell surface [28]. Free cholesterol in nascent HDL particles is subsequently converted to cholesterol esters by the lecithin:cholesterol acyltransferase (LCAT). The absence of functional ABCA1 is the feature of Tangier disease, characterized by a severely impaired lipidation of ApoA1 *via* the ABCA1 pathway, and very low blood levels of HDL [29].

The modulation of intracellular and membrane cholesterol homeostasis has dramatic effects on the early stages of several viral infections [30], [31]. Thus, we hypothesised that stimulation of the ABCA1-mediated cholesterol efflux may influence the course of HCV infection. We provide here the first evidence that pharmacological stimulation of the ABCA1 pathway efficiently inhibits virus-cell entry and decreases virus infection levels in the HCVcc model and in primary human hepatocytes. Our findings highlight ABCA1 as a novel potential target for HCV strategies.

## Materials and Methods

### Cell Infection with HCVcc

The JFH1 HCV strain (genotype 2a) was kindly provided by T. Wakita. Infectious HCV virions were generated as described [32]. Huh7.5 hepatoma cells (kindly provided by C. Rice) were grown as described [33] and were infected with the virus preparation at 10^5^ ffu/ml (focus forming units per millilitre), at an MOI = 0.001–0.01 for 2 h at 37°C. They were then grown further for the indicated time at 37°C.

Primary human hepatocytes were isolated from two adult liver donors after resection for medical reasons and infected with HCVcc as previously described [34].

To culture liver tissue slices, human samples were obtained from adult HCV, HBV and HIV seronegative patients who underwent liver resection in the absence of underlying liver disease. These samples were infected with HCVcc [35].

### Ethics Statement

The French Ministry of Research and Higher Education delivered the authorization N°DC-2008-531 to collect hepatic resection samples from the digestive surgery department and to isolate primary human hepatocytes. The experiments on the human liver slices were performed on surgical samples (from resections of liver metastases), which are considered as “biological waste” and require neither patient nor institutional review board approval. The local Ethics Committee of the Cochin Hospital in Paris, France, approved these experiments.

### Stimulation of ABCA1 Expression

Cell toxicity of LXR agonists GW3965 and TO901317 (Sigma) was determined using the CellTiter-Glo luminescent Cell Viability Assay (Promega). To stimulate ABCA1 expression, Huh7.5 cells or primary hepatocytes were treated with 1–10 µM concentrations of the drugs (diluted in DMSO) for 24 h before infection, or with corresponding dilutions of DMSO. Cells were grown for the indicated time and ABCA1 mRNA levels were analysed by qRT-PCR. ABCA1 protein was determined by Western Blot and quantified using the Odyssey Infrared Imaging system. ABCA1 function was measured using cholesterol efflux assay.

### Quantitative RT-PCR (qRT-PCR)

HCV RNA was determined as previously described [33]. For measurement of the ABCA1 mRNA, 5′-CCTGACCGGGTTGTTCCC-3′ and 5′-TTCTGCCGGATGGTGCTC-3′ primers were used for amplification and 5′-ACATCCTGGGAAAAGACATTCGCTCTGA-3′ (Eurogentec) served as an internal probe. The results were normalized by quantification of HPRT1 or GADPH cellular genes using the HPRT1 Taqman Gene Expression Assay (Life Technology Applied Biosystems) or the GAPDH Control Kit (Eurogentec), respectively.

All assays were performed at least in quadruplicate. Error bars on the histograms represent standard deviation of the mean values from at least quadruplicates.

### Cholesterol Efflux Assay

Control or drug-treated Huh7.5 cells were cholesterol-loaded using 1 μCi/mL [^3^H] cholesterol-labelled SVF (10%) for 24 h in DMEM medium. Cells were then incubated in serum-free medium containing 0.2% BSA with or without 1 μM GW3965 for 16 h. Cellular cholesterol efflux to 25 µg/mL lipid-free ApoA1 (Sigma) was assayed in serum-free medium containing 0.2% BSA for a 4 h chase period, then culture medium was harvested and cleared by centrifugation. Cell-associated radioactivity was determined by extraction in hexane-isopropanol (3∶2), evaporation of the solvent and liquid scintillation counting (Wallac Trilux 1450 Microbeta). The percentage of cholesterol efflux was calculated according to the formula = 100×(medium cpm)/(medium cpm+cell cpm). ApoA1-specific cholesterol efflux was determined by subtracting the cholesterol efflux that occurred in apoA-I-free medium.

### ABCA1 Silencing

Stealth siRNA targeting three different zones of human ABCA1 gene (Life Technologies Applied Biosystems) were used. Huh7.5 cells were transfected with 25 nM ABCA1-specific siRNA or with 25 nM scrambled siRNA (Eurogentec), using the RNAi Max reagent (Invitrogen). At 24 h and 48 h after transfection ABCA1 mRNA levels were determined by qRT-PCR, ABCA1 protein by Western Blot and ABCA1 function by cholesterol efflux assay.

### Cell–to-cell Fusion Assay

The assay was performed as described previously [36]. HEK293T kidney cells (ATCC CRL-1573) were cultured in DMEM supplemented with 10% FBS, 1% non-essential amino acids, 100 U/ml penicillin and 100 µg/ml streptomycin. Cells (2.5×10^5^ cells/well seeded in 35 mm 6-well tissue culture dishes 24 h before transfection) were co-transfected using the calcium phosphate reagent with a plasmid encoding HCV envelope proteins (H77 strain genotype 1a) or Chikungunya envelope encoding-plasmid (ChikV) issued from ChikV E3E1E2 plasmid from the Réunion infectious clone and with an HIV-1 LTR (long terminal repeat) luciferase reporter plasmid (kindly provided by Francoise Bex). After 12 h, transfected HEK293T cells were detached with 0.53 mM EDTA (Invitrogen) and co-cultured (5×10^4^ cells/well) with Huh-7-Tat indicator cells (5×10^4^ cells/well). Co-cultured cells were then incubated with 1 μM of GW3965 or with DMSO. After 24 h, the cells were washed with serum-free DMEM, incubated for 3 min in either pH 7 or pH 5 buffer, (containing 130 mM NaCl, 15 mM sodium citrate, 10 mM MES and 5 mM Hepes), then washed with serum-free DMEM. Cells were next incubated with 1 μM of GW3965 or with DMSO for 48 h. Luciferase activity was measured using a luciferase assay kit (Promega). The experiments were performed several times and results interpreted using student t-test.

### Analysis of Green Fluorescent Protein-Folate Receptor Cell Distribution by Fluorescence Microscopy

Huh7.5 cells were transfected with 0.5 μg of DNA encoding a fusion protein, where GFP is fused to the Glycosylphosphatidyl-inositol-anchor attachment signal of folate receptor (GFP-FR) [37], [38]. Four hours post-transfection cells were treated with 1 μM GW3965 or with a drug solvent (DMSO) that was renewed at 24 h post-transfection. Two days after transfection cells were fixed with 4% PFA containing 0.2% glutaraldehyde. Fluorescence of GFP-FR was visualised with a Zeiss Axioplan 2 microscope at 488 nm (x63 objective).

### Quantification of mRNA of Genes Involved in Lipid Metabolism

Total RNA was extracted using the NucleoSpin RNA II kit (Macherey-Nagel). Reverse transcription and real time qPCR assays were performed as previously described [39] and mRNA levels were normalized compared to those of housekeeping genes (18S, human delta-aminolevulinate synthetase, human alpha-tubulin, human heat shock protein 90 kDa alpha and human hypoxanthine phosphoribosyltransferase 1). Data were expressed as a fold-change in mRNA expression relative to control values.

### Determination of Intracellular Lipids

Quantification of total cellular triglyceride mass was performed as previously described [40]. Free and esterified cholesterol mass was quantified using the Amplex Red cholesterol assay kit (Molecular Probes) [41].

### Cholesterol Replenishment

Control or GW3965-treated Huh7.5 cells were incubated with 20 μg/ml cholesterol:Methyl β Cyclodextrine D (MβCD) complexes (Sigma) for 1 h at 37°C. Cells were extensively washed and analysed for their lipid/cholesterol content or infected with HCV.

### Virus Binding Assay

GW3965 - treated or control Huh7.5 cells were washed with cold medium and incubated with 100 μl of HCV preparation for 2 h at 4°C. Cells were extensively washed to remove the unbound virus, total RNA was extracted and HCV RNA was determined by qRT-PCR.

### HCV Replicon

Huh7 cells harbouring the HCV JFH-1 sub-genomic replicon were kindly provided by J. McLauchlan and grown as described [42]. Replicon cells were treated with 1 µM GW3965 diluted in DMSO for 72 h. Every 24 h the cell culture medium was replaced by fresh medium that contained the drug at the same concentration. As a control, replicon cells were treated in the same way with 0.66 µM cyclosporine A (CsA, Sigma) a known HCV replication inhibitor. Total RNA was extracted from cells every 24 h and HCV RNA assessed by qRT-PCR.

### Determination of HCV Infectivity

An infectivity assay was performed using Huh7.5 cells seeded in 96-well plates and inoculated with serially (ten-fold) diluted HCVcc preparations. After 72 h cells were fixed with 4% paraformaldehyde for 30 min at room temperature and the infected foci were visualized by In-Cell Western assay using human anti-HCV serum and DyLight™ 800 labelled goat anti-Human IgG (KPL, Inc., MD, USA). Fluorescent foci were detected in infected cells using the Odyssey Infrared Imaging System (LI-COR Biosciences, NE, USA). Infectivity was expressed as focus forming units/ml (ffu/ml).

### Ultracentrifugation through an Iodixanol Gradient

Centrifugation was carried out as previously described [33]. A discontinuous 5–50% iodixanol density gradient (OptiPrep) was prepared in a buffer that contained 40 mM HEPES, 270 mM NaCl and 10 mM KCl. The supernatants from infected cells were concentrated using a Vivaspin concentrator (Vivascience) and centrifuged in the gradient for 24 h at 38,000 rpm at 4°C in an SW41Ti rotor of a Beckman ultracentrifuge. Fractions (450 µl) were collected and analysed for the presence of HCV RNA by qRT-PCR. HCV core protein, Apolipoprotein E (ApoE) and Apolipoprotein B (Apo B) were determined by ELISA assays (Mabtech AB, France), and HCV core was quantified by the Chemiluminescent Microparticle Immunoassay (Architect, HCVAg; Abbott Lbs, USA).

### Flow Cytometry

Drug- or solvent -treated Huh7.5 cells were grown in 6-well plates to obtain 1×10^6^ cells/well. Cells were harvested by incubation with Versene, followed by centrifugation and re-suspended in a FACS buffer containing 1% BSA and 0.01% Azide in phosphate-buffered saline (PBS). Cells were then incubated with primary antibodies: monoclonal antibody anti-CD81, rabbit anti-LDL-R antibody (Abcam, Cambridge, UK), or rabbit polyclonal anti SR-BI (kindly provided by T. Huby) for 30 min at 4°C, washed and incubated with APC-conjugated anti-mouse or FITC-conjugated anti-rabbit antibody (BD Pharmigen) for 30 min at 4°C. Cells stained with only secondary antibodies were used as negative controls. Cells were washed with FACS buffer, fixed with 1% paraformaldehyde and analysed by FACSCalibur (BD Bioscience) using FlowJo software (Three stars). A total of 25,000 to 50,000 events were collected per sample.

### Duolink Proximity Ligation Assay

Duolink assay (Sigma-Aldrich) based on proximity ligation technology (PLA) was used to determine the co-localisation of HCV receptors CLDN1 and SR-BI relative to CD81 (<40 nm) in drug-treated or solvent-treated cells. Monoclonal antibodies to CD 81 (Abcam, Cambridge, UK), and rabbit anti-CLDN1 (Abcam, Cambridge, UK) and rabbit polyclonal anti SR-BI (kindly provided by T. Huby) were used in the assay. After signal amplification the slides were examined using fluorescence Zeiss axioplan 2 microscope (x63 objective) at 598 nm. The obtained images were quantitatively analysed using Duolink Image Tool software.

### Determination of SR-BI Function

To investigate cholesterol efflux to HDL *via* the human Scavenger Class B type I Receptor (SR-BI) pathway, SR-BI gene expression was silenced with 50 nM SR-BI specific siRNA. Alternatively 50 nM control siRNA was employed. After 24 h incubation at 37°C, SR-BI knocked-down and control cells were labelled with [^3^H] cholesterol in the presence or absence of 1 μM GW3965. Cholesterol efflux to 50 µg/mL HDL isolated from normolipidemic plasma [43] was assayed during a 4 h chase period in the presence or absence of 1 μM GW3965.

### Western Blot

Cells were lysed with a buffer containing 20 mM Tris-HCl pH 7.5, 150 mM NaCl, 10% glycerol and 1% Triton X-100. After freeze-thawing and sonication, samples of 50 µg total protein were heated for 10 min at 95°C in a sample buffer containing SDS and reducing agent and subjected to electrophoresis in 3–8% Tris-Acetate polyacrylamide. Proteins were then transferred to nitrocellulose membranes and blocked with 5% skim milk in PBS containing 0.1% Tween 20. Blots were then reacted with rabbit anti-CLD1, rabbit anti-OCLN, rabbit anti-pan Cadherin (Abcam Cambridge, UK); rabbit anti-ABCA1 (Novus Bio); or mouse anti-NPC1 (Santa Cruz) as primary antibodies, followed by DyLight 680 conjugated anti-Mouse IgG or DyLight 800 conjugated anti-Rabbit IgG (WWR, France). Blots were quantified using the Odyssey Infrared Imaging System (LI-COR Biosciences, NE, USA).

## Results

### GW3965 Up-regulates ABCA1 Gene Expression and Cholesterol Efflux Function

**Figure 1:**
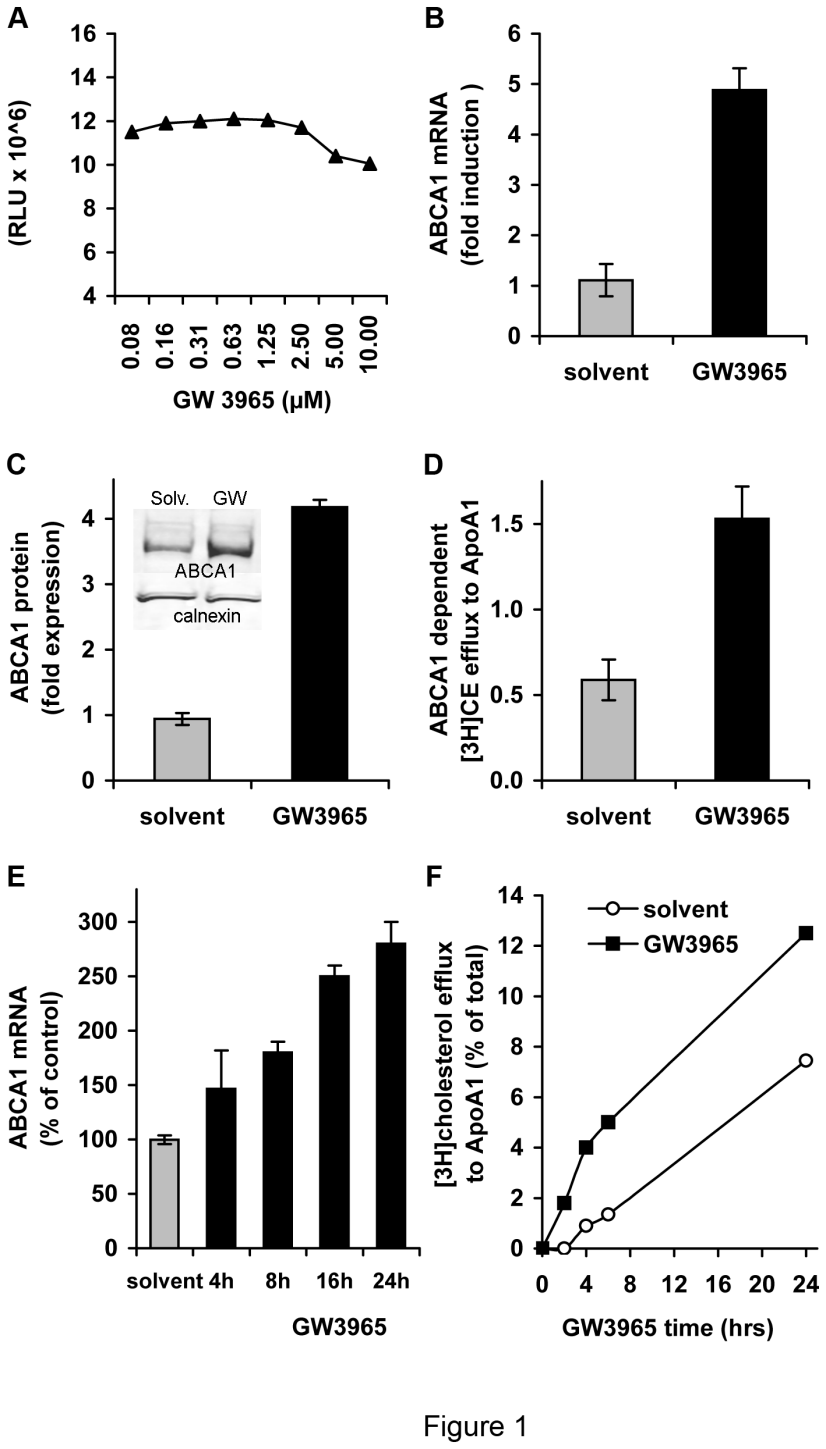
GW3965 treatment up-regulates ABCA1 expression and its cholesterol efflux function. (A) Cell toxicity of GW3965. Huh7.5 cells were cultured in the presence of indicated concentrations of the drug for 24 h. The luminescent signal is expressed in luminescence units (RLU). (B) Up-regulation of ABCA1 mRNA expression by GW3695 treatment. Huh7.5 cells were treated for 24 h with 1 µM GW3695 or drug solvent (DMSO). Then ABCA1 mRNA was determined by qRT-PCR. (C) ABCA1 protein production in drug-stimulated Huh7.5 cells. Cells were treated for 24 h with 1 µM GW3965 and analysed by Western blot (shown in the insert). Protein content in the ABCA1 band (220 kDA) in GW3965-(GW), and DMSO-(solv) treated cells was quantified relative to the calnexin band using the Odyssey Infrared Imaging System. (D) GW3965 stimulation promotes ABCA1-mediated cholesterol efflux to ApoA1. Huh7.5 cells were labelled with [^3^H] cholesterol then incubated with GW3965 or drug solvent. ABCA1-dependent [^3^H] cholesterol efflux was assayed by comparing cell-associated and free radioactivity. (E) Kinetics of ABCA1 gene expression following stimulation of cells with GW3965. Huh7.5 cells were treated with 1 µM GW3965 for the indicated time and ABCA1 mRNA was determined by qRT-PCR. Results were expressed as relative values compared to ABCA1 expression in cells treated with drug solvent. (F) Kinetics of cholesterol efflux in cells stimulated with GW3965. Huh7.5 cells were labelled with [^3^H] cholesterol for 24 h, and incubated for an additional 16 h with 1 μM GW3965 or drug solvent. ABCA1-dependent [^3^H] cholesterol efflux was assayed in the presence of ApoA1 and either GW3965 or solvent for the indicated period of time.

Liver X receptors (LXRα and LXRβ) are ligand-activated transcription factors, which act as cholesterol sensors and control hepatic cholesterol and fatty acid homeostasis [44], [45]. GW3965 is a synthetic compound (LXR agonist) that up-regulates ABCA1 [46]. No appreciable cytotoxicity of the drug for Huh 7.5 cells was observed at a concentration range of 0.08–10 µM (Figure 1A). Treatment of Huh7.5 cells with 1 µM GW3965 for 24 h increased ABCA1 mRNA levels up to 5-fold (Figure 1B). Up-regulation of the ABCA1 gene expression raised ABCA1 protein production (Figure 1C) and enhanced free cholesterol efflux to ApoA1 (Figure 1D). Studies of the kinetics of ABCA1 stimulation showed progressive increase of ABCA1 gene expression until a plateau was reached after 16–24 h (Figure 1E), and gradually augmented cholesterol efflux to ApoA1 (Figure 1F). Thus, 24 h of treatment of cells with the drug was used in further experiments to raise ABCA1 levels.

### Stimulation of ABCA1 Inhibits HCV Infection

**Figure 2:**
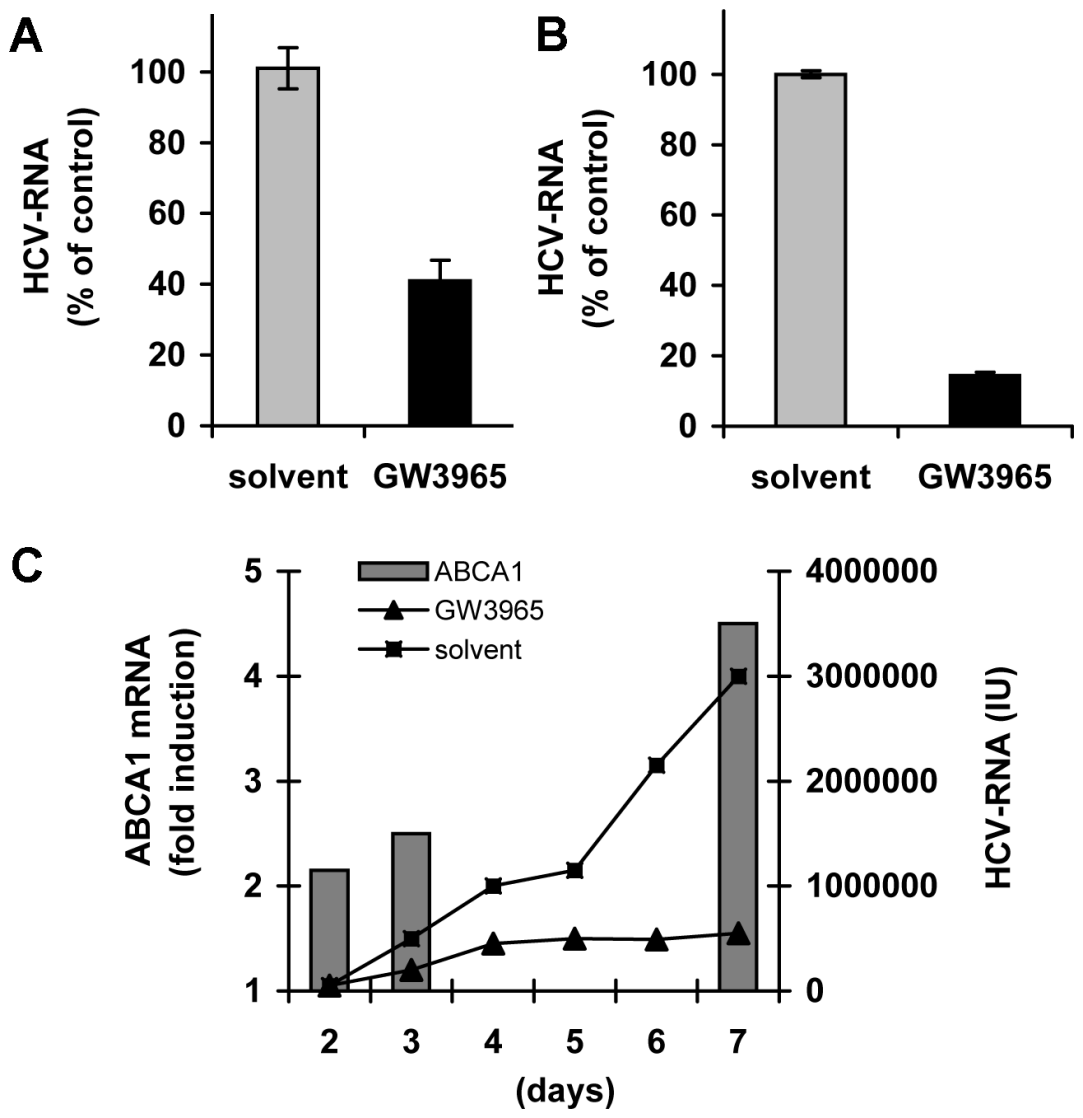
Stimulation of ABCA1 inhibits HCV infection. (A) Reduction of intracellular HCV RNA levels in cells that over-express ABCA1. Huh7.5 cells were pre-treated with 1 µM GW3965 then infected with HCV. Cells were grown for a further 24 h, total RNA was extracted and intracellular HCV RNA was determined by qRT-PCR. Results are expressed as the percentage of HCV RNA relative to that in cells treated with drug solvent prior to infection. (B) Decrease of HCV RNA levels in the supernatant collected from drug-stimulated cells. Huh7.5 cells were pre-treated with 1 µM GW3965 then infected with HCV. After a further 72 h, HCV-RNA in the culture medium was determined by qRT-PCR. Results are expressed as the percentage of HCV RNA secreted from drug-treated cells compared to solvent-treated cells. (C) Effect of GW3965 treatment on long-term HCV infection. Huh 7.5 cells were pre-treated with 1 µM GW3965, infected with HCV and grown for up to 7 days in the presence of the drug. ABCA1 mRNA was determined by qRT-PCR every 24 h and results are expressed as a fold-increase of ABCA1 mRNA compared to solvent-treated cells (grey bars). HCV RNA in the cell supernatant was measured at the same time points by qRT-PCR (line curves for GW3965 treated [filled triangles] or control [filled squares] cells) and is expressed in International Units (IU).

Infection with HCV did not modify ABCA1 gene expression in Huh7.5 cells during 72 h cell growth (not shown). To assess whether stimulation of ABCA1 expression and physiological function would influence the HCV life cycle, Huh7.5 cells were pre-treated with 1 µM GW3965 for 24 h to raise ABCA1 levels and then infected with HCV. Up-regulation of ABCA1 decreased intracellular HCV RNA levels (Figure 2A) at 24 h post infection and significantly reduced the virus production. This was evidenced by the decrease of HCV RNA in the cell supernatant (Figure 2B), which corroborated with a decrease of the concentration of HCV core antigen by 100 fold (not shown). Continuous GW3965 treatment for 7 days substantially reduced the amount of HCV RNA detected in the cell supernatant in parallel with the increase of ABCA1 expression in virus-producing cells (Figure 2C).

TO901317, another synthetic LXR agonist known to be a potent inducer of ABCA1 [47], [48], inhibited HCV infection to the same extent as GW3965 did (not shown). No further decrease of infection was obtained when the two drugs were used simultaneously or in higher concentrations (up to 50 µM). These observations suggested that GW3965 and TO901317 inhibited HCV infection by similar mechanisms.

### ABCA1 Plays a Key Role in the Inhibition of HCV Infection

**Figure 3:**
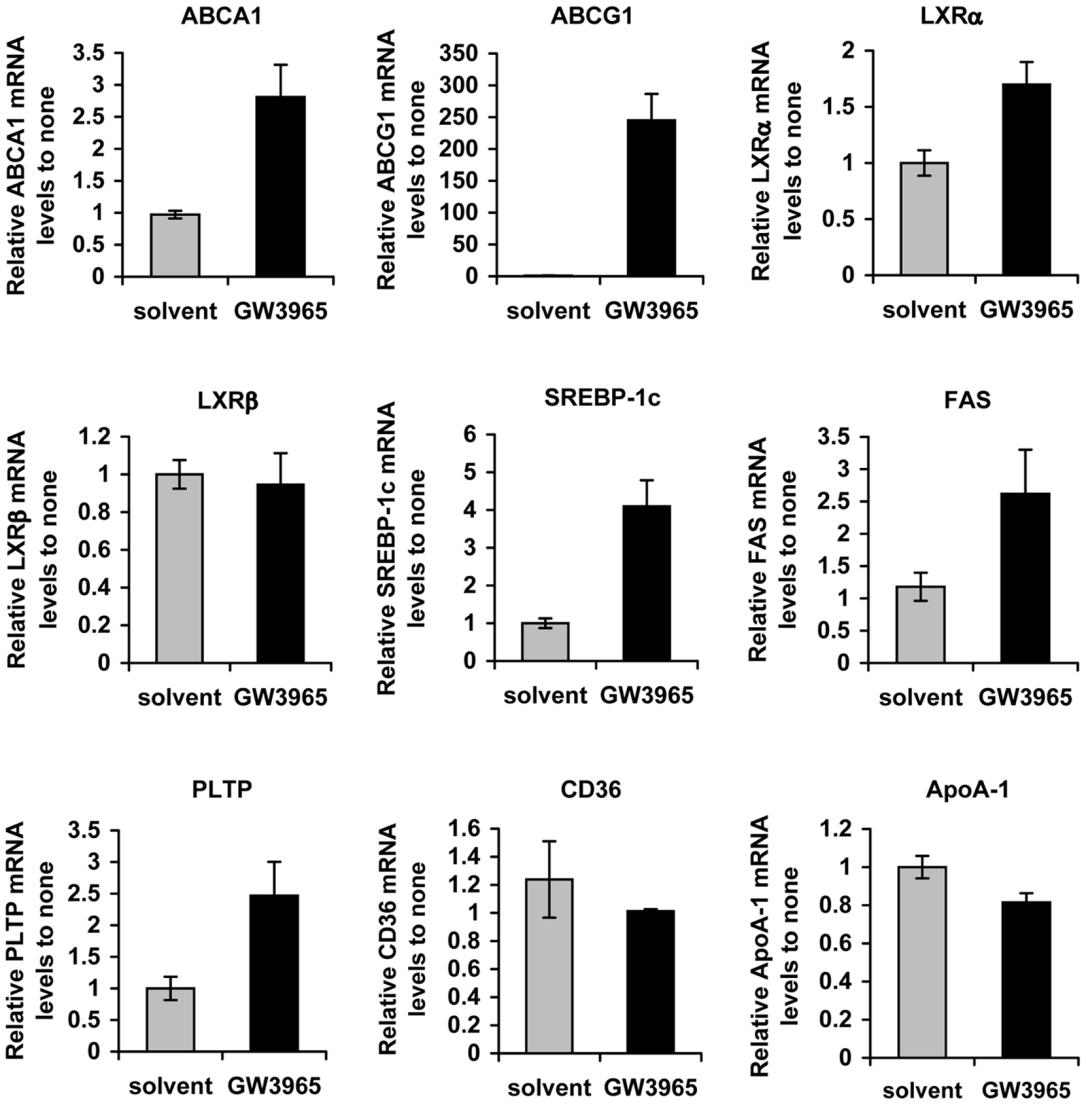
GW3695 treatment modulates expression of genes involved in lipid metabolism. Huh7.5 cells were treated with 1 µM GW3695. Total RNA was extracted from cells and the mRNA levels corresponding to several genes regulating lipoprotein metabolism: ABCA1, ABCG1, nuclear LXRα and LXRβ receptors, a sterol regulatory element binding protein-1c (SREBP-1c), fatty acid synthase (FAS) and phospholipid transfer protein (PLTP), CD36 and ApoA1 were determined by qRT-PCR. The results were normalized to housekeeping genes and compared to the levels of corresponding mRNAs in solvent-treated cells.

Activation of LXRs by their agonists such as GW3965 or TO901317 enhances ABCA1 gene expression but also may affect the expression of other genes that regulate lipid metabolic pathways [45]. Indeed, transcriptomic profiling of Huh7.5 cells treated with GW3965 revealed modified mRNA levels of several genes involved in hepatic lipid metabolism: increased mRNA levels of ABCA1 and ABCG1, nuclear LXRα (but not the LXRβ receptor), a sterol regulatory element binding protein-1c (SREBP-1c), fatty acid synthase (FAS) and phospholipid transfer protein (PLTP). The treatment had no significant effect on mRNA levels of fatty acid transporter CD36 and ApoA1 mRNA (Figure 3).

**Figure 4:**
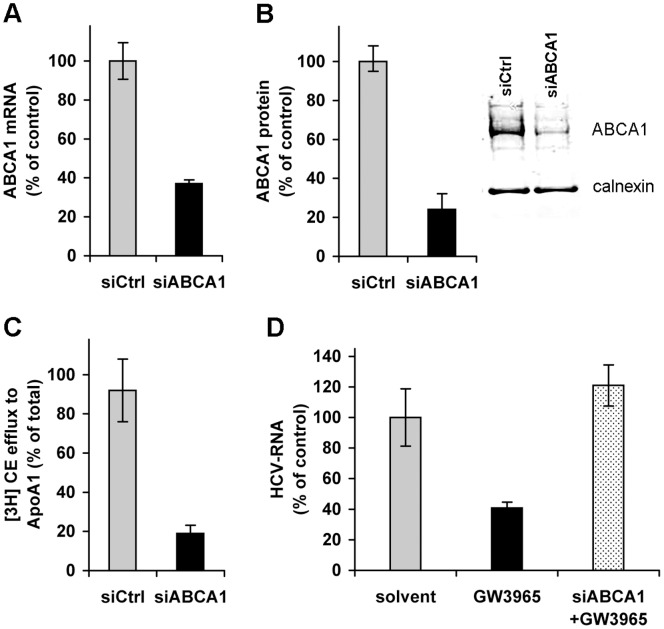
ABCA1 plays a key role in the inhibition of HCV infection. (A) Silencing of ABCA1. Huh7.5 cells were transfected with siRNA that targets ABCA1, or with a control siRNA. Total RNA was extracted after 48 h and ABCA1 mRNA determined by qRT-PCR. Results are expressed as the percentage of ABCA1 mRNA in cells transfected with siRNA targeting ABCA1 relative to mRNA levels in control cells. (B) Decreased ABCA1 protein synthesis in ABCA1-knocked-down cells. Huh7.5 cells were transfected with ABCA1-specific siRNA as above. Cell lysates were subjected to Western blot analysis. Staining of the ABCA1 protein with specific antibodies is shown in the insert. Results are expressed as the ABCA1 protein content in ABCA1-siRNA transfected cells relative to that in control siRNA transfected cells, normalized to calnexin (quantification using the Odyssey Infrared Imaging System). (C) Loss of cholesterol efflux function in ABCA1-silenced cells. Huh7.5 cells were transfected with siRNA that targets ABCA1 or with control siRNA. ABCA1-dependent [^3^H] cholesterol efflux was assayed in the presence of ApoA1. Results are expressed as percentage of cholesterol efflux to ApoA1 in ABCA1 knocked-down cells relative to control si-RNA transfected cells. (D) Silencing of ABCA1 antagonizes GW3965-mediated inhibition of HCV infection. The expression of ABCA1 was reduced by transfection with ABCA1-specific siRNA (as in A) and cells were treated with 1 µM GW3965, infected and grown for a further 24 h. HCV RNA was determined by qRT-PCR. Results are expressed as the percentage of HCV RNA in drug-treated (GW3965) cells or ABCA1-silenced and subsequently GW3965-treated cells (siABCA1+GW3965), compared to solvent-treated cells.

We therefore investigated whether ABCA1 activation was responsible for the inhibition of HCV infection by knocking down the ABCA1 gene. We used three siRNAs that target different regions of ABCA1 gene or a mixture of them to reduce ABCA1 expression. All approaches gave similar rates of ABCA1 reduction (60–80%) as compared to scrambled siRNA. Knocking down of the ABCA1 gene in Huh7.5 cells (Figure 4A) significantly reduced the production of the ABCA1 protein (Figure 4B) and impaired cholesterol efflux to ApoA1 (Figure 4C) as compared to a control siRNA.

To assess whether ABCA1 stimulation was responsible for the inhibition of infection, Huh7.5 cells were transfected with ABCA1 siRNA to knock down its expression (or with scrambled siRNA) and then treated for 24 h with 1 μM GW3965 or solvent. Cells were subsequently infected with HCV and infection levels were evaluated by measuring intracellular HCV RNA at 24 h after infection. Consistent with the requirement of ABCA1 stimulation to induce the inhibitory effect by GW3965 treatment, the drug did not decrease infection levels in ABCA1-silenced cells (Figure 4D).

These results provided evidence that the inhibition of infection was mediated by the over-expressed and fully functional ABCA1.

### GW3695 Treatment Inhibits HCV Cell Entry, but does not Impair other Steps of the Virus-cell Cycle

**Figure 5:**
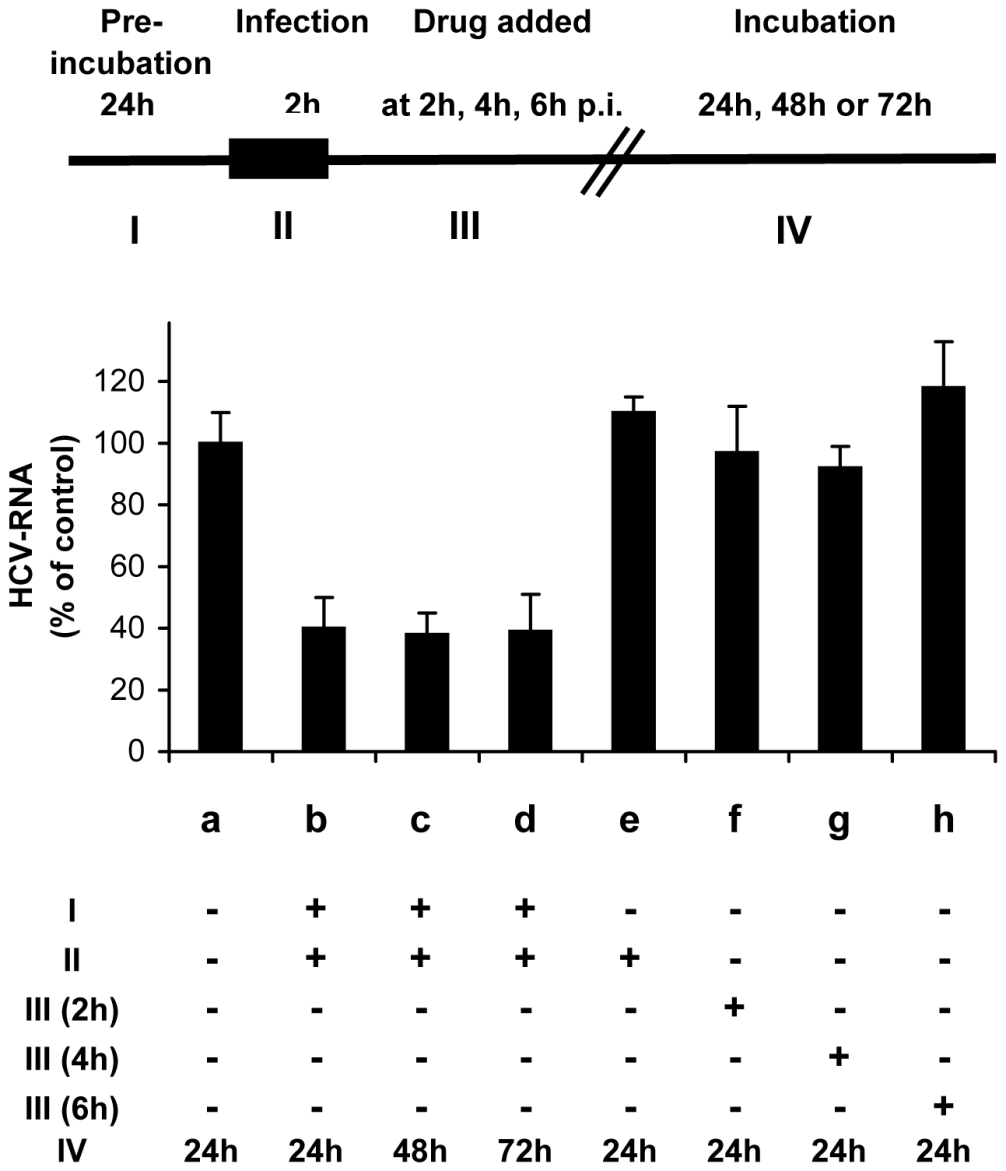
Up-regulation of ABCA1 inhibits HCV cell entry. The effect of GW3965 on the HCV cell cycle was analysed by adding the drug at different time points. A flow-chart is depicted in the upper panel of the graph. RNA in Huh7.5 cells infected in the presence of DMSO is shown in (a); that in cells pre-treated for 24 h with 1 µM GW3965 and infected in the presence of the drug are shown in (b), (c) and (d); results for cells treated with GW3965 during virus inoculation without pre-treatment are shown in (e); those of assays where the drug was added at 2 h, 4 h, or 6 h post-infection are presented in (f), (g) and (h) respectively. For each experiment cells were incubated for the indicated time period after infection (IV). The efficiency of infection was expressed as intracellular HCV RNA measured by qRT-PCR as a per cent of the control (a).

To determine which step of the HCV infection was affected by GW3965 treatment, we analysed the kinetics of activity of this compound by adding the drug at different time points of the virus cycle (depicted in Figure 5). When cells were pre-treated with GW3695 before virus inoculation, and the drug was maintained during infection, intracellular HCV RNA levels decreased at 24 h, 48 h or 72 h post-infection (Figure 5). No inhibition of infection was observed when the drug was added concomitantly with the virus, without cell pre-incubation. These observations provided evidence that GW3965 had no direct detrimental effect on virus structure and infectivity, membrane composition, or fusion events and suggested that raising ABCA1 levels was required to affect infection. Furthermore, no inhibitory effect was noted when the drug was applied at different time points post-infection (Figure 5) implying that pre-treatment of cells with the drug stimulating ABCA1 impaired virus entry, but did not affect later steps of the virus life cycle.

**Figure 6:**
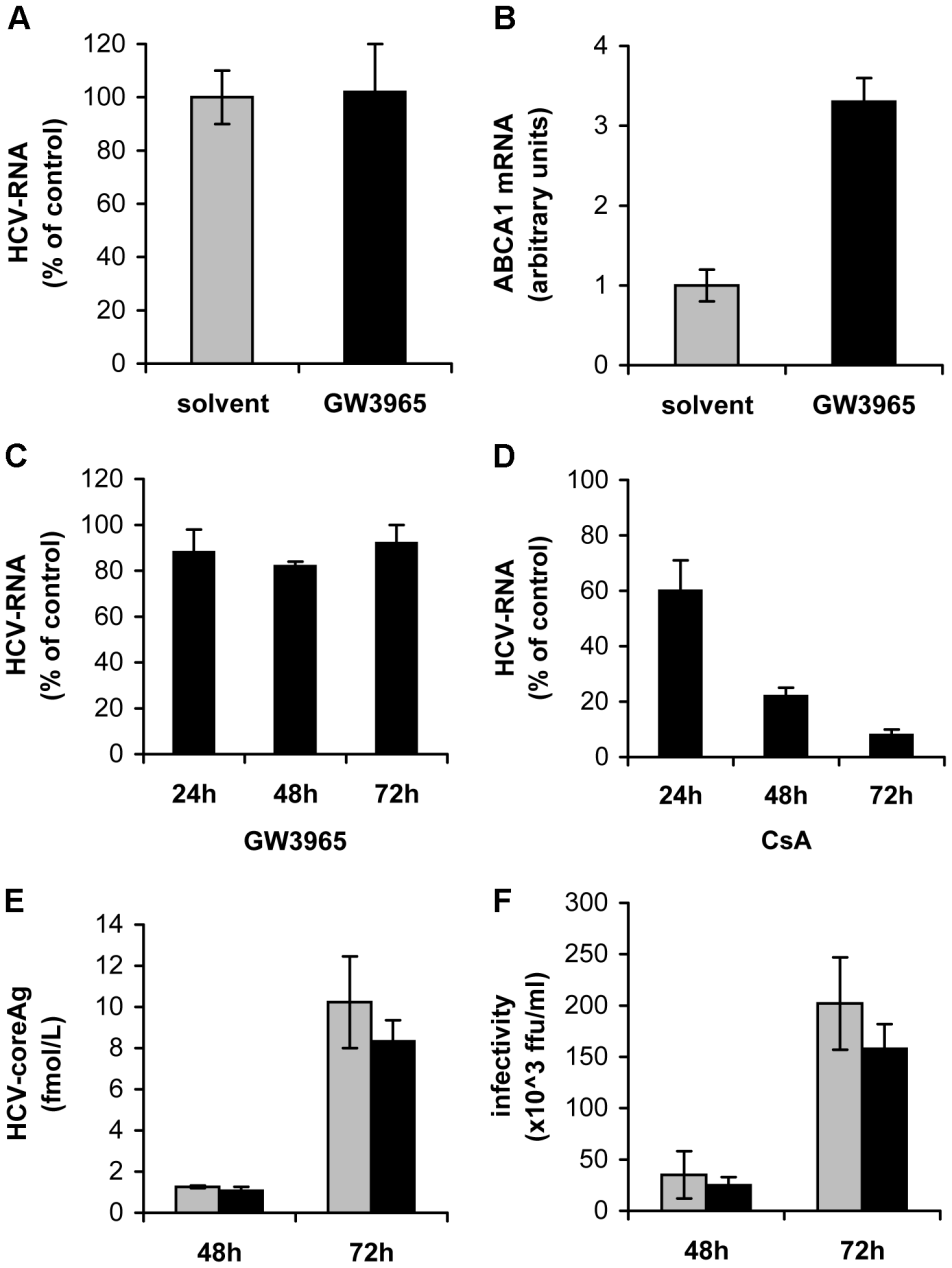
Stimulation of ABCA1 has no impact on HCV cell attachment, HCV RNA replication, assembly/secretion or infectivity of virus particles. (A–B) GW3965 treatment does not affect HCV cell attachment. Huh7.5 cells were pre-treated for 24 h with 1 µM GW3965, to raise ABCA1 levels and tested for their capacity to attach HCV using a “binding assay”. (A) HCV RNA attached to drug pre-treated cells (GW3965) is expressed as per cent relative to HCV RNA attached to cells treated with DMSO (solvent). (B) ABCA1 mRNA levels were determined by corresponding qRT-PCR and expressed in arbitrary units. (C) Stimulation of ABCA1 does not affect HCV RNA replication. Huh7 cells that express the sub-genomic replicon were incubated for 72 h with 1 μM GW3965 or with the equivalent concentration of drug solvent. HCV RNA was quantified by qRT-PCR in drug-treated cells relative to HCV RNA in control replicon cells grown in the presence of drug solvent. (D) HCV RNA replication is inhibited by CsA (control for C). Replicon cells were grown in medium containing 0.67 µM CsA or drug solvent (EtOH) for 72 h. HCV RNA was measured by qRT-PCR every 24 h and results were expressed as the percentage of HCV RNA in drug treated cells relative to HCV RNA content in cells grown in the presence of drug solvent. (E) ABCA1 up-regulation does not affect virus particle assembly/secretion. Huh7.5 cells were infected with HCV and 1 µM GW3965 was applied to cells 2 h post-infection. Every 24 h drug was replenished. HCV core antigen secreted to the cell supernatant was quantified at 48 h and 72 h post infection. Results were normalized with respect to the total protein content in the supernatant and are expressed in fmol/L. (F) Infectivity of virus particles secreted to the cell supernatant from cells treated according to the procedure described in (E) and determined using In Cell Western Blot assay. Results are expressed in ffu/ml.

To assess whether the treatment influenced the initial virus attachment to the cell surface, or later steps in virus-cell entry, Huh7.5 cells were pre-treated with GW3965 for 24 h to stimulate ABCA1 expression. The virus was then added and incubated with cells for 2 h at 4°C. After washings, the cell-bound HCV was quantified by qRT-PCR. These experiments showed that over-expression of ABCA1 after GW3965 treatment (Figure 6A) did not affect virus binding to the cell surface (Figure 6B). Hence, the inhibitory effect induced by over-expressed ABCA1 concerned virus entry events after the binding step.

No inhibition of infection was observed when the drug was added at several time points (2 h, 4 h and 6 h) after infection (Figure 5), suggesting that ABCA1 stimulation does not impair virus replication. This was further confirmed using the sub-genomic HCV replicon model, which permits studies of HCV replication mechanisms without the expression of HCV structural proteins. HCV RNA replication was not affected by GW3965 treatment for 72 h (Figure 6C), but was inhibited, in a time dependent manner by cyclosporine A, known inhibitor of HCV replication, used here as a control (Figure 6D).

Increased ABCA1 expression did not affect mechanisms of assembly/secretion of virus particles. Indeed, when infected cells were continuously treated with GW3965 and the cell medium collected every 24 h (to prevent the effect of ABCA1 stimulation on virus-cell entry) no difference in concentration of HCV core antigen was observed in supernatants collected at 48 h or 72 h post infection, from drug-treated compared to non-treated cells (Figure 6E). The infectivity of virus particles in these cell supernatants was also very similar (Figure 6F).

Collectively, these data demonstrated that stimulation of ABCA1-mediated cholesterol efflux inhibited cell entry step, after virus attachment to the cell surface. However the treatment did not affect other steps of the HCV life cycle and the infectivity of secreted virus particles was unchanged. Accordingly, a decrease of virus production in ABCA1 over-expressing cells was a consequence of reduced virus-cell entry.

### Analysis of HCV Particles Produced in GW3965-treated Cells

**Figure 7:**
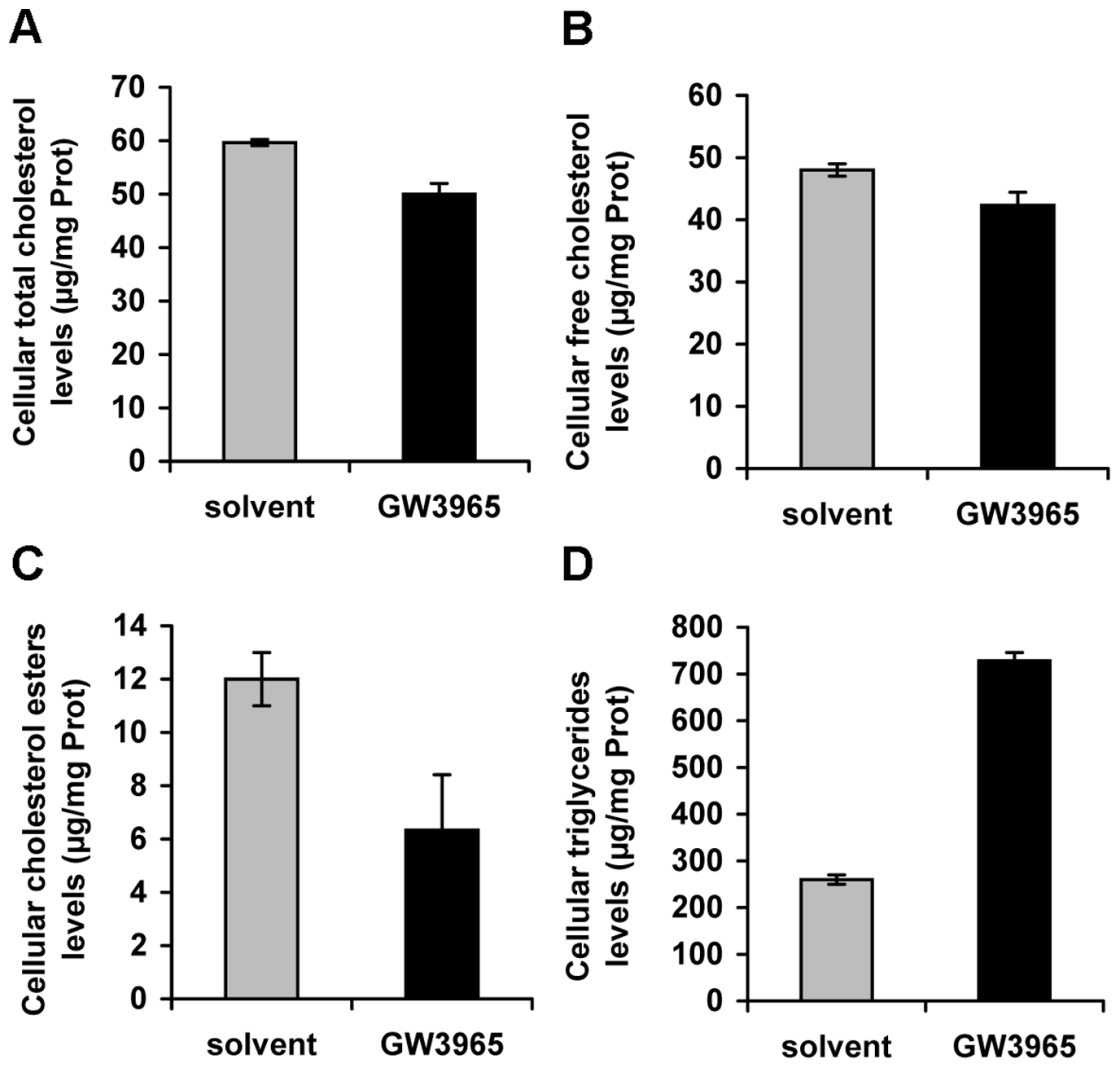
Analysis of cellular lipids in GW3965-treated cells. Huh7.5 cells were treated with 1 μM GW3965 (GW3965) or drug solvent (solvent). Cellular lipids were extracted and (A) cellular total cholesterol, (B) cell-free cholesterol, (C) cholesterol esters and (D) triglyceride levels were quantified and expressed relative to total protein levels.

The increased cellular cholesterol efflux in GW3965 cells did not affect levels of ApoB secreted to the cell supernatant (data not shown) and only slightly affected the total (Figure 7A) and free cholesterol (Figure 7B) content in Huh7.5 cell cultures. Instead, the treatment reduced the concentration of cholesteryl esters (Figure 7C) and increased cellular triglycerides (Figure 7D), as a result of well-known stimulation of lipogenesis *via* LXRs. These changes might potentially affect physical characteristics of the virus particles produced in drug-treated cells.

**Figure 8:**
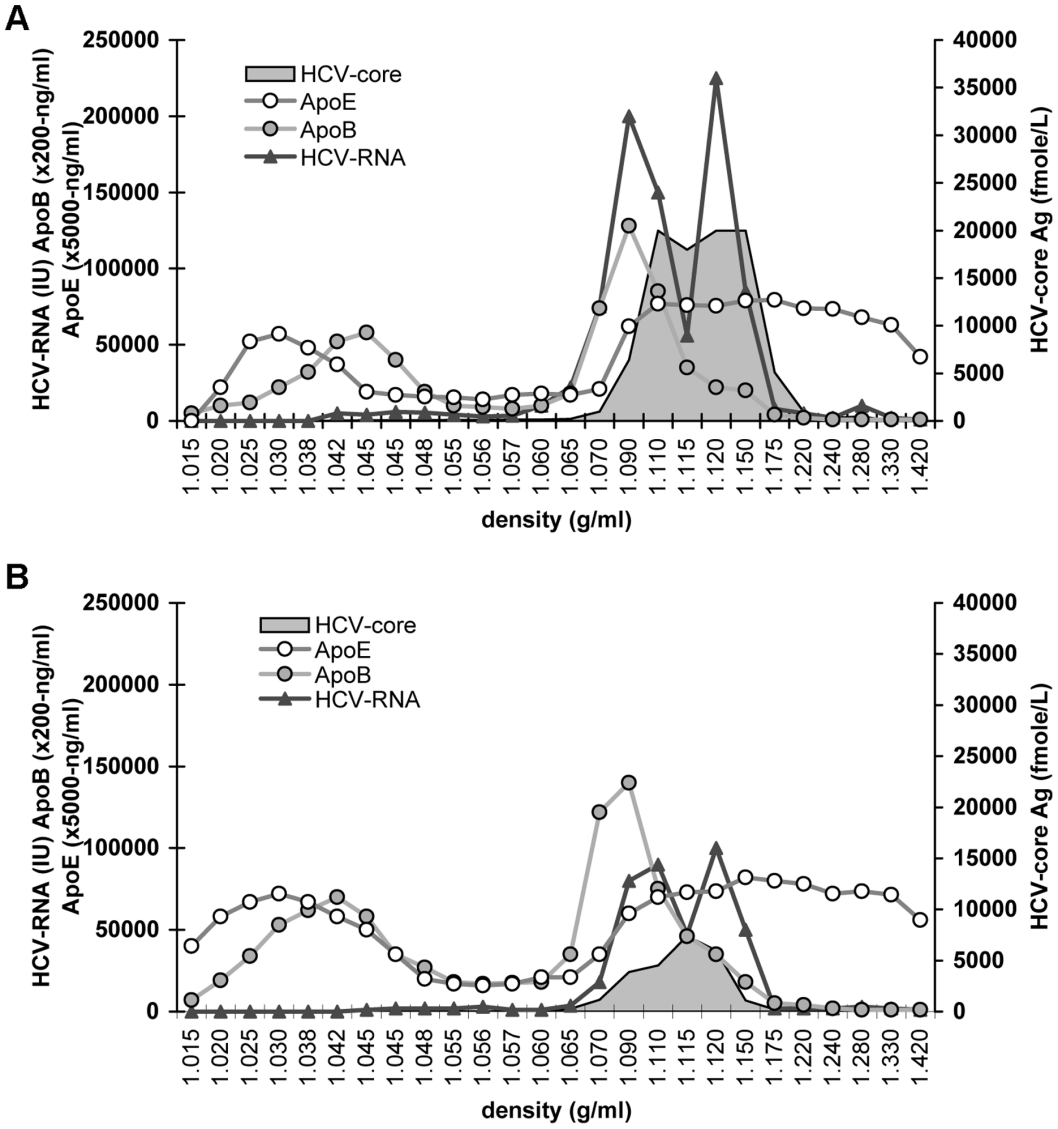
Analysis of HCV particles secreted from cells that over-express ABCA1. Physical properties of the nascent virus particles produced in cells stimulated or not with GW3965 were analysed by centrifugation in iodixanol gradient. Huh7.5 cells were pre-incubated with solvent (panel A) or 1 µM GW3965 (panel B) and the drug was maintained until 72 h post-infection when cell supernatants were collected, concentrated and subjected to gradient centrifugation. HCV RNA in gradient fractions was quantified by qRT-PCR and core antigen, ApoB and ApoE by ELISA assays.

We therefore investigated the properties of virus particles produced in cells continuously stimulated with GW3965 by centrifugation in a discontinuous 5–50% iodixanol gradient. Since significantly less virus was produced and secreted from drug-stimulated compared to non-stimulated cells (shown in Figure 2B) the cell culture medium was concentrated before ultracentrifugation. Nevertheless, in spite of quantitative differences in the levels of virus production, overall properties of viruses produced in the presence or absence of the drug were very similar (Figure 8 A and B). In both gradients HCV RNA was detected in two peaks at a density of 1.1 and 1.12 g/ml. The low-density virus peak co-localised with ApoB and partially with HCV core antigen, which was more abundant in the high density RNA peak.

Thus, stimulation of ABCA1 expression upon GW3965 treatment did not significantly change either infectivity (shown in Figure 6F) or physicochemical properties of the virus particles produced (Figure 8A and B) regardless of the influence of ABCA1 overexpression on the levels of HCV infection and virus production.

### GW3695 does not Change the Expression of HCV Receptors

**Figure 9:**
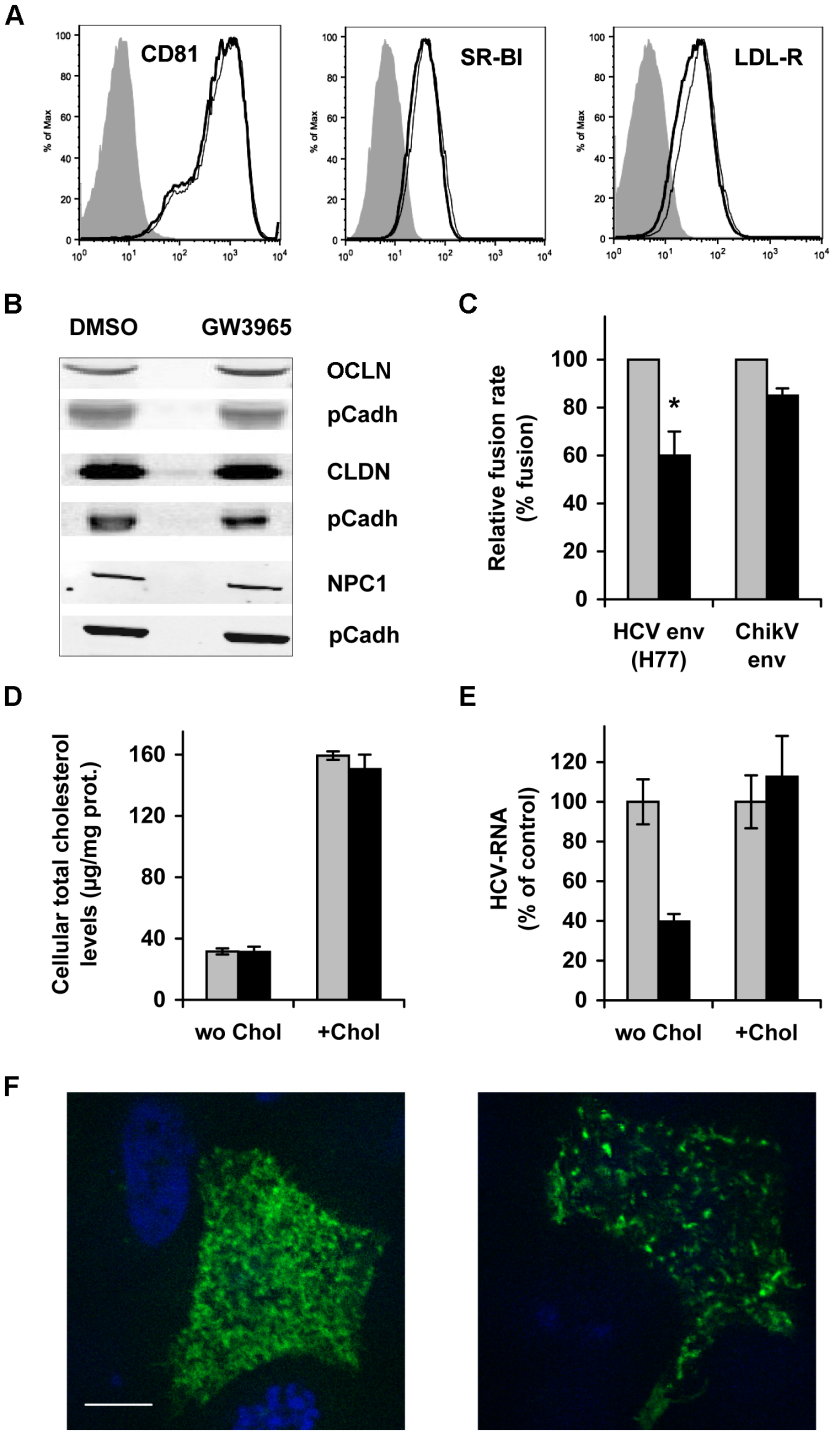
GW3965 treatment does not change the expression of HCV receptors, but affects HCV-cell fusion and modifies lipid raft structure. (A) Analysis of the expression of CD81, SR-BI and LDL-R in Huh7.5 cells treated with 1 μM GW3965 by Flow Cytometry using anti-receptor antibodies. GW3965-stimulated cells are shown by bold lines and non-treated cells by plain lines. Filled histograms represent cells stained with the secondary antibody only. (B) Western Blot assessment of the expression of OCLN, CLDN1 and NPC1 receptor in GW3965-treated cells, compared to solvent-treated cells. Pan Cadherin (pCadh) was used as a loading control. (C) GW3965 treatment inhibits HCV envelope-induced cell fusion. 293T cells that co-express a luciferase marker and the HCV E1–E2 envelope glycoproteins or the Chikungunya virus envelope glycoproteins were co-cultured with Huh7-Tat indicator cells. Co-cultured cells were incubated with 1 µM GW3965 or DMSO and exposed to pH 5. Luciferase activity was measured 72 h later. Data are presented as the fusion rate in the presence of the drug (black bars) relative to fusion in the absence of drug (gray bars), which was considered as 100%. The graph represents the average of 3 independent experiments (*P<0.03). (D–E). Cholesterol loading counteracts the inhibitory effect of ABCA1 over-expression on HCV infection. Huh7.5 cells were stimulated with 1 µM GW3965 to over-express ABCA1, and then incubated with 20 µM cholesterol/MβCD. In (D) the determination of total cellular cholesterol after replenishment of ABCA1 overexpressing cells with cholesterol/MβCD is shown. GW3965-treated cells (black bars) were compared to solvent-treated (grey bars). The infection of Huh7.5 cells after cholesterol supply is shown in (E). Intracellular HCV RNA was determined by qRT-PCR at 24 h post-infection, and is expressed as the percentage of HCV RNA in drug-treated (black bars) compared to solvent-treated (grey bars) cells, supplied (+Chol) or not (wo Chol) with cholesterol. (F) GW3965 treatment modifies plasma membrane organisation and thus the distribution of lipid raft-associated protein. Huh7.5 cells were transfected with DNA encoding the Glycosylphosphatidyl-inositol-anchor attachment signal of the folate receptor fused to GFP (GFP-FR). Cells were subsequently exposed to 1 μM GW3965 or drug solvent (control). Two days post-transfection cells were fixed and GFP-FR fluorescence was visualised using a Zeiss axioplan 2 microscope (x63 objective). Slices of 0.46 µm were acquired. The images shown are a Z projection of 5 slices of the cell surface that face the cell medium. The right panel represents GW3965-stimulated cells and the left control cells. The scale bar corresponds to 10 µm.

We further assessed whether the inhibition of HCV cell entry by ABCA1 over-expression was associated with changes in expression of major HCV receptors. Flow cytometry analyses demonstrated that GW3965 stimulation did not change the global expression levels of CD81, SR-BI, and LDL-R in Huh7.5 cells (Figure 9A). Equally, Western Blot analyses (Figure 9B) confirmed that the expression of NPC1, CLDN1 and OCLN were not modified by the treatment. In addition, ABCA1 stimulation did not modify the cell surface distribution of CD81 relative to SR-BI, or CD81 relative to CLDN1, which interact at the plasma membrane forming HCV receptor complexes [22], [49]. The distance between these pairs of receptors was determined to be <40 nm by the Duolink Proximity Ligation Assay (data not shown). Furthermore, ABCA1 over-expression did not reduce SR-BI specific cholesterol transfer activity to HDL that is required for HCV cell entry [50] (data not shown).

Hence, impaired HCV entry following ABCA1 over-expression was not due to changes in the overall expression of major HCV receptor molecules, or distribution of receptors known to co-localise. It was equally not due to reduced SR-BI cholesterol transfer activity to HDL.

### Stimulation of ABCA1 Affects Virus-cell Fusion

ABCA1 exerts an important influence on the plasma membrane structure, moving cholesterol within the membrane, leading to disorganisation of cholesterol-rich raft domains [51], [52], [53], [54], [55]. These changes might impair virus-cell fusion mechanisms. Using a previously developed assay [36], we found that indeed increased ABCA1 expression upon GW3965 stimulation inhibited cell fusion induced by HCV envelope proteins (*p<0.03), whereas Chikungunya envelope-induced fusion (a control) was not significantly affected (Figure 9C).

These observations suggested the impact of the increased cholesterol efflux on specific membrane rearrangements required for HCV-induced fusion.

### GW3965 Treatment Affects the Organization of Cholesterol Enriched Membrane Microdomains

ABCA1 disorganises cholesterol-rich raft microdomains and redistributes cholesterol/sphingolipids from raft to non-raft domains making it available for ApoA1 and facilitating HDL production [51], [52], [53], [54], [55] Supplying cells that over-express ABCA1 with exogenous cholesterol should redistribute membrane cholesterol between these domains and thus inverse the effect of ABCA1 [56].

To assess whether ABCA1-stimulated inhibition of HCV entry might be reversed by the cholesterol supply, we incubated cells that over-expressed ABCA1 with cholesterol/MβCD complexes. Cholesterol/MβCD–treatment increased total cellular cholesterol content (Figure 9D) and completely reversed the inhibitory effect of ABCA1 stimulation on HCV cell entry (Figure 9E).

These data suggested that the increased cholesterol efflux *via* ABCA1 induced remodelling of the cholesterol-rich lipid raft microdomains and accordingly affected virus-induced fusion. The inhibitory effect could be reversed by an exogenous cholesterol supply, providing evidence that restriction of HCV infection was induced by changes of cholesterol content/distribution in membrane regions essential for virus-cell entry.

To confirm that indeed GW3965 treatment affected cholesterol-enriched membrane microdomains (lipid rafts) we analysed the plasma membrane distribution of a raft-associated Glycosylphosphatidyl-inositol-anchor attachment signal of the folate receptor (GFP-FR). Glycosylphosphatidyl-inositol (GPI)-anchored proteins are cholesterol-dependent for their plasma membrane organization and thus can be used as lipid raft markers [37], [57]. Fluorescence microscopy analysis demonstrated that, while in untreated cells GFP-FR was homogenously distributed at the plasma membrane, in GW3965-treated cells GFP-FR expression was drastically different from the control (Figure 9 F and G). Thus, ABCA1 stimulation affected the organization of cholesterol enriched membrane microdomains.

### GW3965 Inhibits HCV Infection of Primary Human Hepatocytes

Since Huh7.5 cells currently used as a model for studies of HCV infection have several differences in lipid metabolism compared to primary human hepatocytes, we investigated the effect of ABCA1 up-regulation on HCV infection of human hepatocytes in primary culture [34] or HCV infection of human liver slices [35], the two available models that are permissive for a full HCV life cycle and display normal lipid/lipoprotein metabolic pathways.

**Figure 10:**
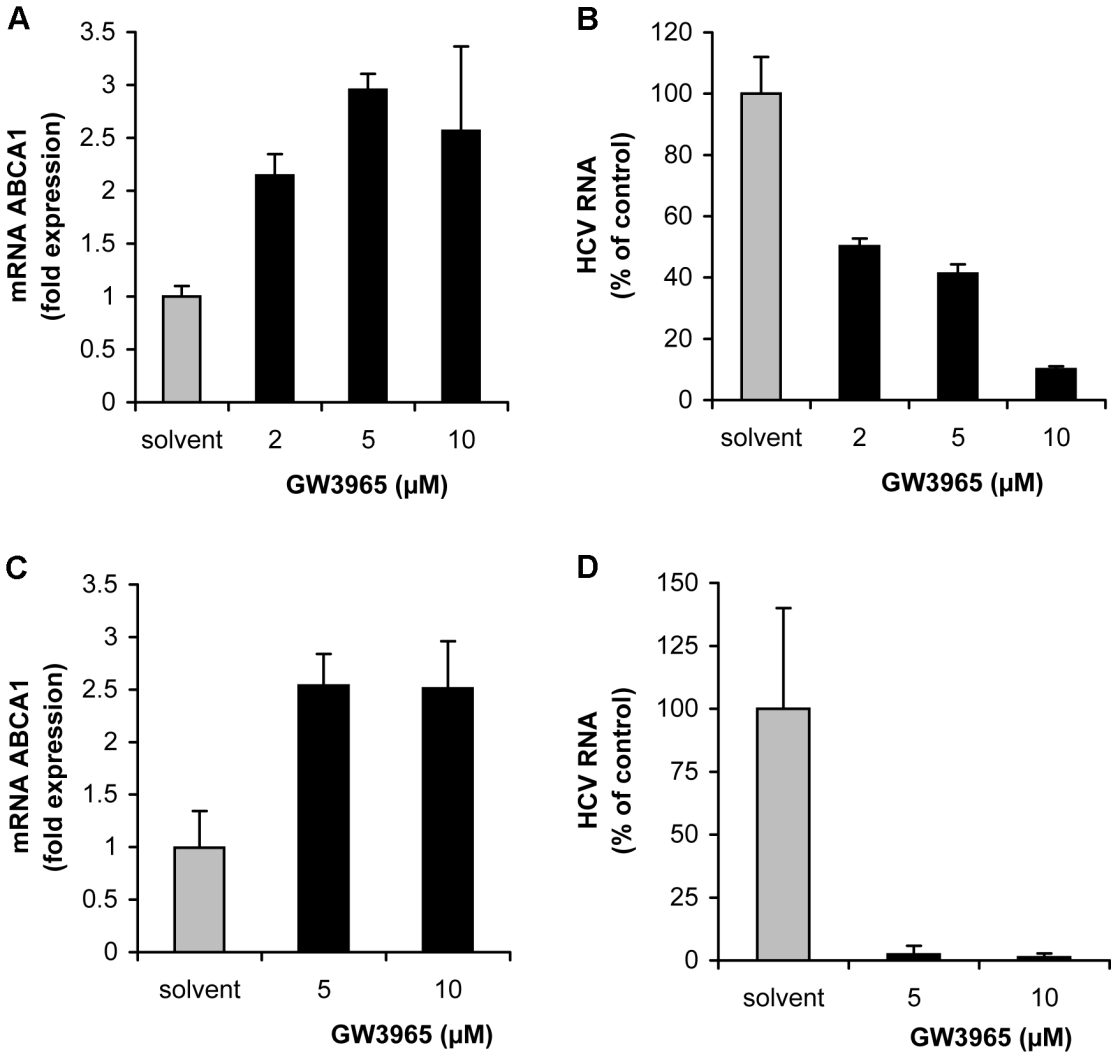
Over-expression of ABCA1 inhibits HCV infection of primary human hepatocytes and human liver slices. (A–B) Inhibition of HCV infection of primary human hepatocytes. (A) Primary human hepatocytes were treated with 2–10 μM GW3965 (non-toxic concentrations for cells) or with drug solvent, prior to HCV infection. Twenty-four hours post-infection, ABCA1 mRNA was determined by qRT-PCR and expressed in arbitrary units, taking into account ABCA1 levels in liver cells pre-treated with the drug. (B) GW3965-treated and solvent-treated primary human hepatocytes were inoculated with HCV. After 24 h, intracellular HCV RNA was quantified by qRT-qPCR. The efficiency of infection in drug pre-treated cells was expressed as the percentage of infection compared to solvent-treated cells. (C–D) ABCA1 over-expression inhibits HCV infection of human liver slices. Human liver slices were cultured for 24 h, treated with 5 or 10 μM GW3965 or with DMSO before infection with HCVcc. At 24 h post-infection, total RNA was extracted and ABCA1 mRNA (C) and HCV RNA (D) were quantified by corresponding qRT-PCR assays and expressed as the percentage of RNA compared to the values obtained for solvent-treated cells.

First, hepatocytes obtained from two liver donors were pre-treated with various concentrations of GW3965 (2–10 µM). Cells were then infected with HCVcc and grown for 24 h or 48 h and HCV RNA was determined by qRT-PCR. As shown in Figure 10 (A–B) ABCA1 up-regulation was associated with a dose dependent decrease of intracellular HCV RNA levels.

A substantial inhibition of HCV infection was also observed in the model of primary human liver slices cultures. Cultures of human liver slices were established with differentiation status and were treated with 5 μM and 10 μM concentrations of GW3965 or with drug solvent, for 24 h before infection with HCVcc. These drug concentrations were selected on the basis of results showing that they were not toxic for cells and were required to raise ABCA1 levels.

HCV RNA levels decreased at least by two logs in drug-treated cells, compared to non-treated cells, while ABCA1 gene expression concomitantly increased 2.5 fold over the time course of the experiment (Figure 10 C and D).

Altogether these data provided evidence that pharmacological stimulation of ABCA1 expression efficiently inhibited HCV infection of primary hepatocytes.

## Discussion

In this work, we provide the first demonstration that pharmacological stimulation of ABCA1 gene expression and its cholesterol efflux function with the LXR agonist GW3965 impairs HCV infection, acting on virus protein mediated-host cell fusion. The inhibitory mechanism was associated with re-organisation of cholesterol-rich membrane microdomains (lipid rafts). Stimulation of ABCA1 expression also efficiently inhibited infection of human hepatocytes in primary culture (the natural HCV target cell) and isolated human liver slices.

LXRs are ligand-activated transcription factors, which maintain cholesterol homeostasis. LXR agonists increase ABCA1 expression but also modulate several other genes that regulate lipid metabolic pathways [58], [59]. Indeed, we found that GW3965 affected the expression of several genes involved in hepatic lipid metabolism. Nevertheless, silencing of the ABCA1 gene and reducing its protein expression and specific efflux function reversed the inhibitory effect of GW3965, supporting the key role of ABCA1 in the inhibition of HCV infection.

In hepatocytes, the specific cholesterol efflux function and nascent HDL formation only depends on ABCA1, whereas ABCG1, another cholesterol transporter, acts in synergy with ABCA1 to promote cellular cholesterol efflux to preformed nascent HDL particles. SR-BI, one of the main HCV receptors, contributes neither to cholesterol efflux to ApoA1 nor to HDL formation [60]. Thus the specific cholesterol efflux function to Apo A1 activated by the treatment with GW3965 or TO90317 was due to ABCA1 activity.

Stimulation of ABCA1 by these drugs impaired HCV cell entry by acting on virus protein-mediated cell fusion, presumably blocking virus nucleocapsid release into the cytosol. GW3965 did not directly affect the virus particle or cell membrane structure, since adding the drug during virus inoculation did not reduce its infectivity. Instead, inhibition of infection required ABCA1 over-expression and activation of its function during several hours. GW3965 did not influence either virus binding to the cell surface or HCV RNA replication.

Up-regulation of ABCA1-mediated hepatic cholesterol efflux might decrease hepatic VLDL secretion [61]. In our study GW3965 treatment did not affect either levels of ApoA1 in the cell supernatant, or virus assembly/secretion mechanisms. It did not change physicochemical properties or infectivity of the virus particles produced in drug-treated cells. Thus the decrease in HCV production and secretion was due to impaired virus-cell entry.

The inhibition of HCV cell entry *via* stimulation of the ABCA1 pathway was not associated with changes in overall expression of HCV receptors: LDL-R [8], HSPG [62], CD81 [63], SR-BI [64], NPC1 receptor [65] and tight junction molecules CLDN1 [66] and OCLN [67] required for HCV cell entry. Nevertheless, besides its reliance on the expression of receptor molecules, initiation of a productive HCV infection depends on the cholesterol content of the target cell membrane [22]. Indeed, cholesterol constitutes an essential component of lipid-rich membrane microdomains, organised parts of the plasma membrane. These membrane domains compartmentalise/segregate proteins and lipids and thus play a key role in virus entry, either influencing clustering of receptors or acting on virus-cell fusion [23], [68].

Depletion of the membrane cholesterol with MβCD inhibited HCV infection [22] showing for the first time that cholesterol-rich raft environments are likely to serve as portals for HCV entry. While MβCD disrupts lipid rafts by depleting cells of cholesterol with high cytotoxicity [69], ABCA1 stimulation leads to the activation of the physiological cholesterol efflux pathway that does not affect total and free cellular cholesterol content, and has low cytotoxicity. Whereas MβCD treatment resulted in important changes of cell surface expression of CD81 and SR-BI [22] and OCLN [70], such changes were not observed in our study in cells over-expressing ABCA1. Indeed, using the Duo Link assay we found that the cell surface distribution of SR-BI and CLDN1 relative to CD81, the HCV receptors supposed to co-localise [22], [49] was not modified in GW3965-stimulated cells (distance <40 nm).

Up-regulation of ABCA1 leads to a perturbation of cholesterol packaging in cell membranes and induces changes in its distribution in the lipid rafts or between raft and non-raft microdomains [51], [53], [54], [56]. Such modifications might impair HCV-host cell fusion, as shown in our fusion assay, either by influencing distribution of lipids and proteins or by changing membrane fluidity [23].

Indeed, cholesterol facilitates HCV-mediated fusion dependent upon the presence of functional E1 and E2 proteins [36]. The fusion proteins act in common with lipid and cholesterol assemblies at the virus-cell fusion step. Lipids, mainly glycerophospholipids, sphingolipids and sterols, contribute through their physical, mechanical and/or chemical properties, whereas cholesterol plays a role through its preferential partitioning into rafts or its binding affinity for certain viral envelope proteins. Cholesterol-rich microdomains are implicated in the entry of many virus species such as Ebola and Marburg viruses, Vaccinia virus, murine hepatitis virus, lymphocytic choriomeningitis virus and Herpes Simplex Virus. Indeed, cholesterol dispersion or reorganisation of lipid microdomains using drugs such as filipin and nystatin reduced virus entry, although virion attachment was unaffected. Since all these viruses enter host cell via cholesterol-rich microdomains, these observations suggested drug action on a fusion step [71].

Several studies underline the fact that ABCA1 expression does not alter the overall cellular cholesterol content or its subcellular distribution [53]. The current model proposes that ABCA1 exerts an influence on plasma membrane structure, modifying the organisation of cholesterol-rich microdomains by redistributing cholesterol/sphingolipids to non-raft domains that facilitates ApoA1 interaction and efflux [51], [53], [54], [56]. In agreement with this notion, in our study activation of the ABCA1 pathway had only a minor effect on total and free cholesterol content in Huh7.5 cells. Instead, stimulation of ABCA1 modified the cholesterol distribution in cell membranes. Indeed, lipid raft-dependent localisation of GFP-FR in membranes was dramatically changed in GW3965-treated cells. These changes most probably disturbed virus-cell entry, confirming that the integrity of cholesterol-rich membrane microdomains is essential for the initiation of a productive HCV infection.

Supplying a surplus of exogenous cholesterol to GW3965-treated cells overturned the inhibitory effect on HCV cell entry, in accordance with the notion that adding exogenous cholesterol to cells that over-express ABCA1 tightens lipid packaging in raft and non-raft microdomains, while decreasing ABCA1-dependent cholesterol efflux [56].

HCV (JFH-1 strain) infection of the Huh7.5 cell line is currently used for studies of the HCV life cycle and the evaluation of HCV inhibitors. Nevertheless, this *in vitro* cell culture system, based on transformed and poorly differentiated hepatoma cells, has several limitations, especially concerning lipoprotein metabolism [72]. Thus, we confirmed our findings in primary culture of human hepatocytes and in human liver slices, which maintain the 3D structure and gene expression of the liver with normal lipid/lipoprotein metabolic pathways. We provide evidence that stimulation of ABCA1 expression with GW3965 inhibits HCV infection in primary hepatocytes even more efficiently than in Huh7.5 cells.

HIV infection depends on membrane cholesterol for virus assembly, budding as well as cell entry, which also requires lipid rafts [73]. Strikingly, *via* its protein Nef, HIV down-regulates ABCA1 expression and impairs cholesterol efflux activity to infect its target cell [74]. ABCA1 overexpression achieved using the TO901317 LXR agonist impaired HIV infection; accordingly, pharmacological stimulation of ABCA1 has been proposed as an approach to inhibit HIV infection [75].

ABCA1-mediated cholesterol efflux leads to the formation of HDL, a lipoprotein preventing arteriosclerosis and cardiovascular disease progression. New drugs are under development that should reduce the side effects found for LXR agonists such as an increase in triglyceride levels [76], [77] also observed in our study. Moreover, cholesterol efflux function has been linked to anti-tumour activity, and thus ABCA1 is considered as a tumour-suppressor molecule [78].

Our study suggests that in addition to other known beneficial roles, ABCA1 emerges as a potential target to better control HCV infection.
